# Effects of Mandibular Canine Intrusion Obtained Using Cantilever Versus Bone Anchorage: A Comparative Finite Element Study

**DOI:** 10.7759/cureus.27548

**Published:** 2022-08-01

**Authors:** Afshan S Waremani, Nausheer Ahmed

**Affiliations:** 1 Orthodontics and Dentofacial Orthopedics, Government Dental College and Research Institute Bangalore, Bengaluru, IND

**Keywords:** mini-implant, fem, intrusion, cantilever, arch wire

## Abstract

Background

This study was conducted to assess and compare the effects of mandibular canine intrusion obtained using a cantilever having different toe-in bends and with mini-implants using the three-dimensional (3D) finite element method (FEM).

Methodology

3D models of the mandibular right quadrant were created using FEM. Brackets and molar tubes were also modeled. In the first model, the mandibular canine intrusion was produced using a cantilever loop with compensatory toe-in bends (0°, 4°, 6°, and 8°). In another model, the intrusion was done using two mini-implants. Force was applied using an elastic chain. The amount of intrusion and the associated labial tipping of canine that occurred in both the models was assessed and compared using FEM analysis.

Results

The pure intrusion of the canine was produced by the 6° toe-in bend of the cantilever. The labial tipping of the canine was also reduced. The highest amount of periodontal ligament stress was observed around the canine root with a 0° toe-in bend. In the posterior segment, the molar displayed a slight tendency for extrusion and distal crown tipping.

Conclusions

The intrusion mechanics using a cantilever simulated in this study may achieve pure mandibular canine intrusion with minimal labial tipping when a compensatory toe-in of 6° is incorporated into the cantilever.

## Introduction

A deep overbite is a malocclusion commonly encountered in orthodontic practice [1]. A deep bite can be categorized as dentoalveolar in nature or skeletal due to the growth of the jaws [2]. At the most basic level of analysis, the skeletal and dental components that appear to be consequential in affecting overbite change are (1) maxillary skeletal displacement, (2) mandibular skeletal displacement, (3) maxillary dental change, and (4) mandibular dental change [3]. The exaggerated curve of Spee has been shown to play a main role in developing dental deep bites [3,4]. A deep bite can be treated by various methods, of which mandibular incisor intrusion is the most suitable treatment for adults with normal incisor and gingival display and a normal or high mandibular plane angle [5]. Ricketts et al. described a technique using the utility arch and gently tying an elastic band from the canine bracket to the utility arch [6]. Intrusion refers to the apical movement of the geometric center of the root (centroid) with respect to the occlusal plane or a plane based on the long axis of the tooth. Labial tipping of an incisor around its centroid produces pseudo-intrusion as it influences the vertical incisal edge position [7]. Most deep bite cases have anatomically extruded mandibular canines, and the treatment plan often involves the intrusion of the incisors and the canines [8]. Lower incisor intrusion can be accomplished using different arches, a three-piece intrusion arch, or a utility arch, with the disadvantage being incisor proclination and unwanted distal tipping of anchor teeth [9]. Weiland et al. showed that segmented arch mechanics can produce genuine intrusion of the incisors with little vertical effect in the molar area in adult patients [10]. Approximately half of the patients with deep bites have clinically extruded mandibular canines. The orthodontic intrusion of the six anterior teeth together can cause undesirable effects in the posterior segment; hence, a segmented method of intrusion should be used [11]. Marcotte suggested the use of a cantilever from the auxiliary tube of the first molar to the canine bracket slot. Burstone also described a method for the individual intrusion of the canines with a slight compensatory toe-in bend to deliver a lingual force to control the buccal tipping of the canine [11].

Miniscrew anchorage is also used for tooth intrusion because it can apply a low, continuous force without causing reciprocal movements of the other teeth [12]. When one wishes to intrude a tooth while keeping its axial inclination, the buccal insertion of two mini-implants on either side is recommended [13]. In orthodontics, the finite element method (FEM) has been used to evaluate the risk of adverse events during technical procedures to verify and simulate different loading systems and evaluate the effects on the dentoalveolar structures [14]. Uncontrolled canine intrusion in the treatment of deep bites may lead to buccal crown tipping and thus increase the chances of orthodontic treatment relapse [15]. This may increase the risk of gingival recession or bone resorption. Hence, there is a need to improve the clinical method for intruding the mandibular canines while adequately leveling the curve of Spee [11]. The objective of this study was to use the FEM to compare the effects of the intrusion of mandibular canine obtained using a cantilever with different compensatory buccolingual activations versus mini-implants.

## Materials and methods

**Figure 1:**
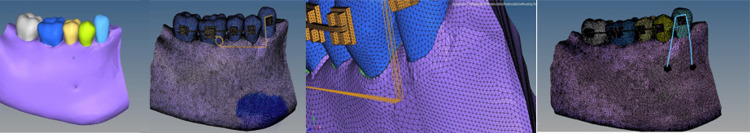
Left to right: A model of the bone from the canine to the second molar and teeth with 1.5 mm offset of the canine. Bracket and wire with four different toe-in bends of the cantilever, that is, 0°, 4°, 6°, and 8° (zero from the left side), assembly with the cantilever arrangement. The model with an elastic chain placed from mini-implants to the canine.

Two geometric models of the mandibular arch from the right second permanent molar to the right permanent canine were created through computed tomography (CT) scan and converted to a three-dimensional (3D) step file format through a reverse engineering technique (ANSYS) (Figure 1). The teeth were modified with proper crown-to-root ratio and leveled. The canine with its alveolar bone was extruded by 1.5 mm in simulation.

The brackets, tubes (0.022 × 0.028-inch slot), and the base wire were assumed to be composed of stainless steel. In the first model (Figure 1), a cantilever (0.017 × 0.025 inches) made of titanium-molybdenum alloy (elastic modulus = 69 GPa; Poisson’s ratio = 0.3) with a helix of 3 mm in diameter was simulated (Table 1). One end of the cantilever was fitted inside the first molar auxiliary tube. The horizontal segment extended mesially up to the interproximal contact point between the first premolar and the canine. At this point, a 90° bend was modeled occlusally, comprising a vertical segment that ended at the level of the upper portion of the canine bracket. Finally, another 90° bend was made to generate the final segment of the cantilever, which was in contact with the canine (Figure 1). The second model consisted of two self-drilling mini-screws of 6 mm in length and 1.6 mm in diameter (Ti6 Al4,113.8 GPa elastic modulus, Poisson’s coefficient = 0.3) inserted buccally at a 90° angulation, in the interdental area, one on either side of the mandibular canine (Figure 1) [16]. A straight 0.019 × 0.025-inch stainless steel archwire was simulated alongside the canine’s buccal surface immediately below the bracket to prevent undesirable lingual root torque. Intrusive force to the tooth was applied using an elastic chain from the mini-screws to the canine bracket. The first model was further subdivided as follows: Model 1(a) - model having a cantilever loop with a toe-in of 0° for the intrusion of the canine; Model 1(b) - model having a cantilever loop with a toe-in of 4° for the intrusion of the canine; Model 1(c) - model having a cantilever loop with a toe-in of 6° for the intrusion of the canine; Model 1(d) - model having a cantilever loop with a toe-in of 8° for the intrusion of the canine (toe-in bends were placed immediately after the helix, as depicted in Figure 1); and Model 2 - model with two mini-screws placed buccally and mesial and distal to the canine for the intrusion.

**Table 1:** Material properties of the members.

Member	Elastic modulus	Poisson’s ratio
Tooth	20 GPa	0.3
Periodontal ligament	0.71 MPa	0.4
Bone	345 MPa	0.3
Stainless-steel wire	200 GPa	0.3
Titanium molybdenum alloy	69 GPa	0.3
Implant	110 GPa	0.33

Data representation

**Figure 2:**
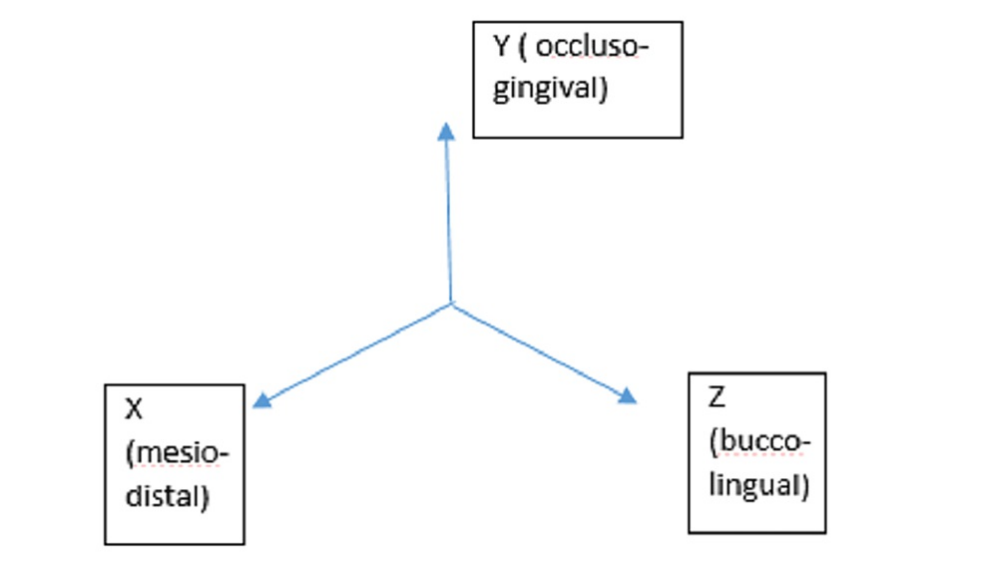
Illustration of the direction.

In this study, all the interactions present between the brackets and wires were determined using beam elements. The remaining contacts between the elements of different objects had rigid contact interactions, in which phases from the different materials remain without relative displacement between them. The following three reference axes (X, Y, Z) (Figure 2) having the mandibular canine as the reference point were used for cantilever activation: (1) X-axis representing the mesiodistal aspect; (2) Y-axis representing the occluso-gingival direction; and (3) Z-axis representing the buccolingual aspect.

Experimental conditions

In the first model, a cantilever was used for the intrusion of the canine (Figure 1). It was placed from the molar tube to the canine bracket and pre-activated with a 35° tip-back. This applied a force of 0.02 N on the X-axis (mesiodistally) and 0.37 N (occluso-gingivally) on the Y-axis of the canine. The following compensatory toe-in bends were simulated in this experiment: 0°, 4°, 6°, and 8°, with each applying a force of 0.01 N, 0.052 N, 0.082 N, and 0.12 N on the Z-axis (Figure 1; Table 2). The activation of each simulated compensatory toe-in was equal to the magnitude of the force in the X-axis, inferred from the visualization. Therefore, the force variation was only on the X-axis. Moreover, the resultant counter-effect produced by the cantilever with different toe-ins on the anchor molar causing its movement along the Y-axis and Z-axis was determined [11].

**Table 2:** Amount of force on the X-axis produced with the toe-ins tested.

Toe-in	Force in the Z-axis (N)
0°	0.010
4°	0.052
6°	0.082
8°	0.12

In the second model, two mini-screws were placed at angulation of 90° buccally in the interdental bone on both the mesial and the distal sides of the canine, and an intrusive force of 0.147 N was applied onto the tooth using an elastic chain extending from the mini-screws to the canine bracket.

The amount of pure intrusion was measured by the movement of the tooth along the Y-axis, and the amount of buccal crown tipping that occurs due to different compensatory toe-in bends of the cantilever was measured along the Z-axis. In addition, the counter-effect on the posterior anchorage system and stress changes in the alveolar bone and periodontal ligament surrounding the canine and the molar tooth were assessed. In the second model, the canine intrusion was achieved using two mini-screws placed on either side of the target tooth with an elastic chain from the mini-screws to the tooth. The results obtained from these two models were evaluated and compared using the 3D finite element analysis using ANSYS software.

## Results

The values of pure intrusion produced by two finite element models were obtained. Stresses and displacements were calculated and represented in colored bands, with different colors representing different stress levels and different values for maxillary molar displacements. The red-colored area of the spectrum indicates maximum principal stress, followed by orange, yellow, green, and blue representing the reducing levels of stress. Two nodes, the tip of the buccal cusp (crest node = no.128046) and the apex of the root (root node = no. 129160) were selected for evaluating the movement of the canine. When intrusive forces were applied, the amount of displacement along the Y-axis was measured and tabulated (Table 3).

**Table 3:** Amount of intrusion of the crest node and root node displacement along the Y-axis.

	0°	4°	6°	8°	Mini-implant
Crest node	-0.949 µmm	-3.056 µmm	-5.486 µmm	-7.31 µmm	-1.9 µmm
Root node	-0.184 µmm	-2.456 µmm	-5.027 µmm	-7.37 µmm	-1.6 µmm

**Figure 3:**
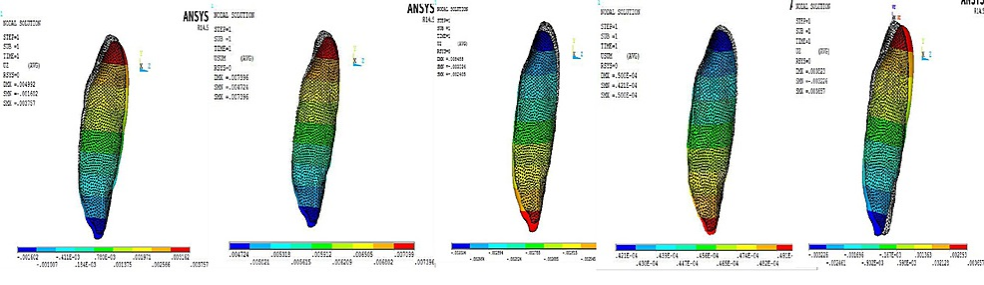
Displacement of the root and crest nodes in 0°, 4°, 6°, and 8° cantilever models and implant model (left to right).

**Figure 4:**
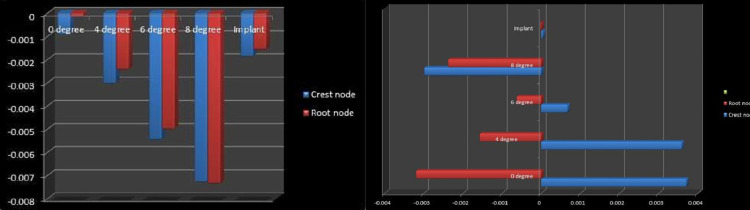
Left to right: Amount of intrusion of crest node and root node displacement along the Y-axis. Labial/lingual tipping (displacement along the Z-axis).

The displacement of the canine along the Y-axis due to the 0°, 4°, 6°, and 8° toe-in bend of the cantilever is represented in Figure 3. The displacement of the canine due to forces applied by the mini-implant is represented in Figure 3. The amount of intrusion obtained by the 8° toe-in bend of the cantilever was found to be the highest among all models, and the least value was produced by the cantilever with the 0° toe-in bend (Figure 4).

The amount of labial/lingual tipping is represented in Table 4. Figure 4 illustrates the movement of the crest and root node. The results showed that in the mini-implant model the least amount of tipping of the canine tooth occurred on the application of intrusive forces, whereas the maximum amount of labial tipping of the canine was seen with the model having a cantilever with a 0° toe in bend (Figure 4).

**Table 4:** Labial/lingual movements of crest and root node displacement along the Z-axis.

	0°	4°	6°	8°	Mini-implant
Crest node	3.71 µmm	3.59 µmm	0.675 µmm	-3.01 µmm	0.0485 µmm
Root node	-3.219 µmm	-1.59 µmm	-0.65 µmm	-2.4 µmm	-0.0365 µmm

**Figure 5:**
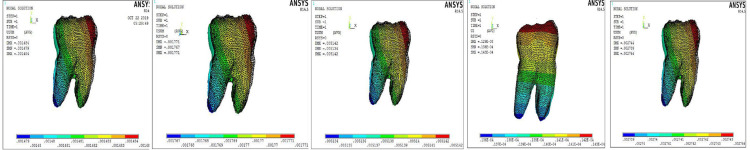
Effects on the molar (0°, 4°, 6°, and 8° toe-in models) and the implant model (left to right).

The counteracting effects on the molar produced by the cantilever are illustrated in Figure 5. The molar tooth in all the models showed slight extrusion and distal tipping of the crest node. However, the implant model did not display any significant molar counter-effects (Figure 5). The periodontal stresses around the root of the canine were evaluated and are tabulated in Table 5. The 0° toe-in cantilever model displayed the maximum stress in the periodontium of the canine, followed by the cantilever model with the 6°, 8°, and 4° toe-in a decreasing order (Table 5). The stresses occurring in the alveolar bone around the canine were also evaluated and are tabulated in Table 6. The maximum stress values were obtained in the implant model (0.0039 MPa, Table 6), while the least amount of alveolar bone stress was seen with the cantilever with the 0° toe-in bend (0.0016 MPa, Table 6).

**Table 5:** Stresses in the canine periodontium.

	0°	4°	6°	8°	Mini-implant
Stress (MPa)	0.002443	0.00194	0.002361	0.002069	0.340E-03

**Table 6:** Alveolar bone stress around the canine.

	0°	4°	6°	8°	Mini-implant
Stress (MPa)	0.001603	0.002215	0.003581	0.003192	0.003994

## Discussion

A deep bite is a complex orthodontic problem and its correction is an important part of treatment due to the deleterious effects on the temporomandibular joint, periodontal health, and facial aesthetics [9]. Deep bite cases generally have anatomically extruded mandibular canines, and the treatment plan often involves the intrusion of the anterior teeth [8,11]. Several studies have been conducted to intrude the mandibular canine individually, but are often faced with the problems of unwanted labial tipping of the concerned tooth. One technique was described by Ricketts et al. and involved using the utility arch [6]. Another technique was reported by Marcotte and Burstone [8] who suggested the use of a cantilever from the auxiliary tube of the first molar to the canine bracket slot. However, these techniques do not include a method for controlling unwanted tipping [8,11]. Hence, in our study, a cantilever loop was used to intrude the mandibular canine. The cantilever was tested with 0°, 4°, 6°, and 8° toe-in bends which were incorporated to determine the effectiveness of each toe-in bend in controlling unwanted labial tipping of the mandibular canine on the application of intrusive forces. A study by de Araújo et al. showed that pure cuspid intrusion can be achieved by the use of elastic forces applied from two mini-implants placed on either side of the labial surface of the canine root. The unwanted buccal tipping was prevented by the placement of a rigid 0.019 × 0.025-inch stainless steel wire on the labial surface of the crown just below the bracket [13]. A few studies have been conducted to determine the true intrusion of the canine. Caballero et al. in an FEA study used a cantilever loop with different toe-in bends to intrude the mandibular canine and showed that the 6° toe-in bend produced pure intrusion of the canine tooth [11]. The study also showed that the amount of tipping decreased with an increase in the toe-in bend. The anchor molar displayed a tendency for extrusion and distal tipping [11].

In our study, the intrusion produced by the 8° toe-in bend of the cantilever was found to be the highest among all models, and the least value was produced by the cantilever with the 0° toe-in bend (Table 3). The amount of pure intrusion increased with an increase in the degree of toe-in bend, and the amount of labial tipping of the canine decreased with an increase in toe-in up to 6°; however, the 8° toe-in bend produced lingual tipping of the tooth. Both the implant model and the 6° toe-in model showed almost pure intrusion with the least amount of labial tipping, although the intrusion values produced by the 6° toe-in of the cantilever model were higher. The maximum tipping was present in the cantilever with a 0° toe-in bend, indicating that incorporating a toe-in reduces the tendency of labial tipping of the concerned tooth (Table 4). This is in accordance with the study by Caballero et al. [11]. In the posterior segment, the anchor molar showed a tendency for extrusion and distal tipping in all the cantilever models (Figure 5), whereas this was negligible in the implant model (Figure 5). Tanne et al. (1987), in a 3D FEM study, reported a cervical margin stress of 0.012 N/mm^2^ when a lingually directed tipping force of 1 N was applied to the center of a mandibular premolar model [17]. A study by Jones et al. showed that the maximum strains recorded in the surrounding alveolar bone were 35 times less than for the periodontal ligament. This FEM model validated that the periodontal ligament is the main mediator of orthodontic tooth movement [18]. Geramy (2002) reported the stress produced in the periodontal membrane by orthodontic load and that tipping caused increased stress in the cervical margin of the periodontal membrane, and in case of intrusive movements, at the apical and subapical levels [19]. In the present study, among the models, the maximum periodontal stress was seen with the cantilever with the 0° toe-in bend followed by the 6°, 8°, and 4° toe-in models (Table 5). Polat-Ozsoy et al. showed that mini-screw mechanics produce pure intrusion of the incisors [20]. The simulations of this finite element study showed that a significant amount of labial crown tipping occurs when intrusive forces are applied to a canine tooth without any labiolingual control. In such cases, the control of tipping movements of a tooth is extremely important as it can lead to various unwanted problems. Increased tipping of the tooth can lead to an increased risk of gingival recession, periodontal problems, alveolar bone loss, and abfractions of the tooth. A study by Bernhardt et al. evaluated the multifactorial causes that lead to abfractions and concluded that gingival recessions are associated with the genesis of abfractions [21]. In the present FEM study, the results showed that the amount of labial tipping tendency of the tooth decreased when a toe-in bend was added to the cantilever. The 6° toe-in bend of the cantilever was sufficient to produce almost pure intrusion of canine with very less amount of tipping tendency. This is in accordance with the results seen in the study by Caballero et al. The forces applied from the mini-implants also produced pure intrusion of the canine with negligible tipping, but the amount of intrusion produced was less compared to the cantilever model. The posterior segment was consolidated and served as a rigid anchorage system in cantilever models; hence, only a slight tendency for extrusion and distal tipping was noted in the molar [11]. The periodontal stresses were maximum in the model with the 0° toe-in bend and the least in the mini-implant model. Although the stresses seen with the 6° toe in the model were slightly on the higher side, it produced a good amount of pure intrusion with almost negligible labiolingual tipping making it the appliance of choice when the true intrusion of a canine is desired.

## Conclusions

This FEM study showed that, among all the models, the cantilever model with the 6° toe-in produced a pure intrusion with minimal labial tipping. The study proved that the incorporation of compensatory toe-in bends in a cantilever is necessary to prevent undesirable labial or lingual crown tipping of the mandibular canines on the application of intrusive forces. Moreover, intrusion of the mandibular canine with less labial tipping can be achieved using two mini-implants. The labial tipping is reduced with the use of a 0.019 × 0.025-inch stainless steel wire placed just below the bracket of the tooth. In the posterior anchorage segment, the first molar displayed a tendency for extrusion and distal crown tipping in all the cantilever models, whereas the effects were negligible in the mini-implant model. Most of the registered periodontal stresses were around the canine root. The stress was the highest in the model that was devoid of any compensatory toe in bend.

In this FEM study, idealized tooth models were used to simulate the conditions, and the results obtained from this study were based on a one-time simulated tooth movement. In day-to-day practice when dealing with patients having different tooth morphologies, differences in periodontal health, alveolar bone conditions, and biological reactions, the resultant effects may vary. The results obtained in this study may serve as a future reference guide for further customization of different compensatory bends with cantilevers and influence the mechanics with mini-implants. Further clinical studies are needed to confirm the results of this study.
